# Development and validation a simple model for identify malignant ascites

**DOI:** 10.7150/ijms.53743

**Published:** 2021-03-03

**Authors:** Ying-Yun Guo, Xiu-Lan Peng, Na Zhan, Shan Tian, Jiao Li, Wei-Guo Dong

**Affiliations:** 1 Department of Gastroenterology, Renmin Hospital of Wuhan University, Wuhan, Hubei, 430060, China.; 2 Department of Oncology, The Fifth Hospital of Wuhan, Wuhan, Hubei, 430050, China.; 3 Department of Pathology, Renmin Hospital of Wuhan University, Wuhan, Hubei, 430060, China.

**Keywords:** diagnosis, differential, ascites, carcinoma, logistic models

## Abstract

The differential diagnosis of benign ascites and malignant ascites is incredibly challenging for clinicians. This research aimed to develop a user-friendly predictive model to discriminate malignant ascites from non-malignant ascites through easy-to-obtain clinical parameters. All patients with new-onset ascites fluid were recruited from January 2014 to December 2018. The medical records of 317 patients with ascites for various reasons in Renmin Hospital of Wuhan University were collected and reviewed retrospectively. Thirty-six parameters were included and selected using univariate logistic regression, multivariate logistic regression, and receiver operating characteristic (ROC) curve analyses to establish a mathematical model for differential diagnosis, and its diagnostic performance was validated in the other groups. Age, cholesterol, hypersensitivity C-reactive protein (hs-CRP) in serum, ascitic fluid adenosine deaminase (AF ADA), ascitic fluid lactate dehydrogenase (AF LDH) involvement in a 5-marker model. With a cut-off level of 0.83, the sensitivity, specificity, accuracy, and area under the ROC of the model for identifying malignant ascites in the development dataset were 84.7%, 88.8%, 87.6%, and 0.874 (95% confidence interval [CI], 0.822-0.926), respectively, and 80.9%, 82.6%, 81.5%, and 0.863 (95% CI,0.817-0.913) in the validation dataset, respectively. The diagnostic model has a similar high diagnostic performance in both the development and validation datasets. The mathematical diagnostic model based on the five markers is a user-friendly method to differentiate malignant ascites from benign ascites with high efficiency.

## Introduction

Ascites fluid is a common clinical syndrome, which can be divided into benign and malignant ascites for various reasons. When disorders, such as heart failure, renal failure, liver cirrhosis, and hypoproteinemia, affect the peritoneum or protein balance, the pathological fluid, called ascites, accumulates in the peritoneal cavity 1. Malignant ascites is the result of cancer and account for nearly 10% of all ascites cases occurring in related to various tumors, especially breast, ovary, stomach, pancreas, and colon cancer, and presents a challenging clinical problem in some cases 2. However, the distinction between the two types of ascites fluid is not only the basis of diagnosis but also a prerequisite for formulating a treatment plan.

Differential diagnosis of benign and malignant ascites can rely on certain precise findings such as appropriate ascites fluid serum analysis, cytological examination, laparoscopy, and the symptoms of the patients. Previous studies have shown that several biochemical indicators, such as total protein 3, serum-ascites albumin gradient (SAAG) 4, and various tumor markers 5, 6, are vital for the differential diagnosis of benign and malignant ascites. However, they all have their own limitations. The use of a predominant beneficial tool, SAAG, is also a matter of debate in etiological diagnosis 7. The cytology analysis positive rate is only 30-50%, although it has proven to be the gold standard for identifying malignant ascites 8. The positive rate of peritoneal biopsy is relatively high, but it is not easy for patients to accept because of its invasive nature. Furthermore, the procedures that lead to a definitive diagnosis could result in a delay that threatens the adequate management of the disease and its outcomes 9. The search for novel biochemical markers in serum and/or ascitic fluid (AF) is still under investigation. Yun et al. 10 reported that exosomal miRNAs offer a novel biomarker for discriminating gastric cancer ascites from non-malignant ascites. However, like other new biomarkers, this assay is not accessible, especially in hospitals in developing countries. Hence, continuous work should be taken to design a simple, cost-effective, and less invasive method to provide accurate information about the etiology of ascites.

In this study, to address the disadvantages of previous studies, we aimed to explore the diagnostic power of easily attainable demographic features and laboratory indicators of patients with ascites to establish a diagnostic model for identifying malignant ascites with high efficiency.

## Materials and Methods

### Patient selection

We performed a retrospective review of the medical data of all patients with new-onset ascites admitted to Renmin Hospital of Wuhan University from January 2014 to December 2018. The participants in this study were recruited with the following criteria: (a) patients with newly developed ascites, (b) patients who underwent diagnostic abdominal paracentesis and (c) patients who were informed and agreed to participate in the study. Individuals were excluded from the study based on the following criteria: (a) patients who had received peritoneal dialysis treatment, (b) patients with bloody ascites caused by trauma or surgery, (c) patients with ascites of unknown etiology, (d) patients with missing information, and (e) patients with signs of sepsis.

**Figure 1:**
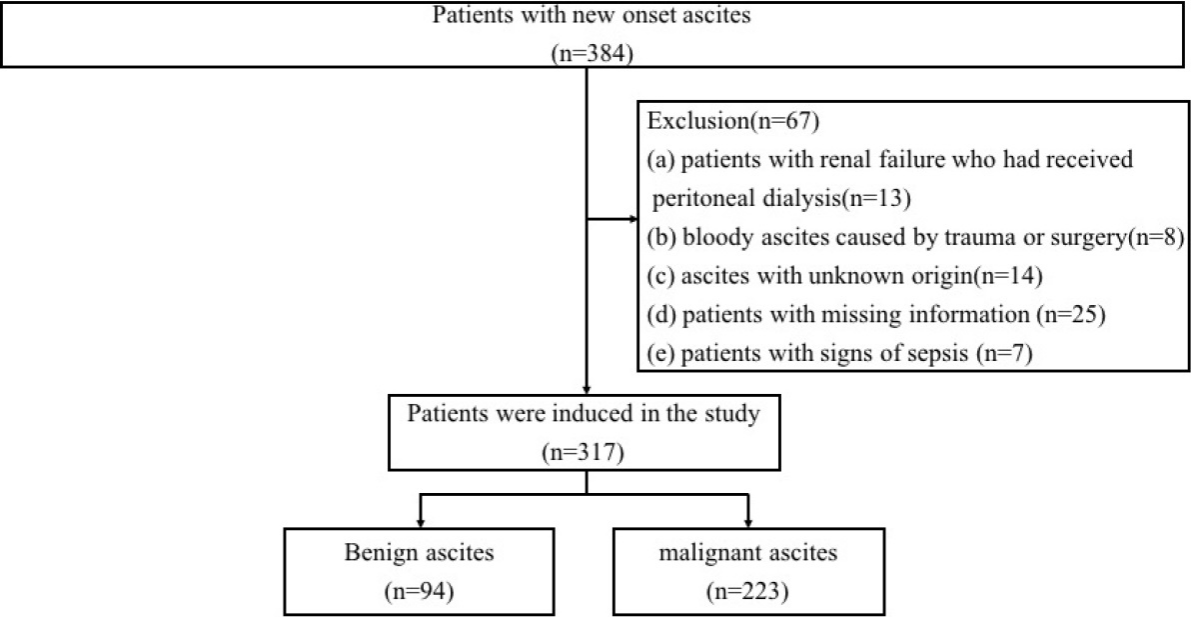
Flowchart of patient inclusion.

In total, 317 participants were enrolled in our study (94 patients with benign ascites and 223 patients with malignant ascites), and 67 subjects were excluded. The flow of patient selection is shown in Figure 1. Ethical approval for this study was obtained from the Ethics Committee of Renmin Hospital of Wuhan University in Hubei Province (WDRY2019-K014). All participants signed written informed consent after the study protocol was thoroughly explained, and this study was conducted in accordance with the Helsinki Declaration.

### Data collection

All patients underwent abdominal paracentesis and blood withdrawal in the fasting state before initiating any treatment, such as administration of intravenous fluids or diuretics or chemotherapy. The medical records of each patient were reviewed in a structured manner to obtain the patients' demographic characteristics and laboratory data. In serum laboratory evaluations, white blood cells (WBCs), neutrophil (Neu), lymphocyte (Lym), red blood cells, hemoglobin, red blood cell distribution width-standard deviation (PDW-SD), platelets, platelet distribution width (PDW), alkaline phosphatase (ALP), total protein (TP), albumin, chloride, total cholesterol (TCh), hypersensitivity C-reactive protein (hs-CRP), C-reactive protein (CRP), lactate dehydrogenase (LDH), alpha-fetoprotein (AFP), cancer antigen 125 (CA125), cancer antigen19-9 (CA19-9), and carcinoembryonic antigen (CEA) during the initial diagnostic period were evaluated.

In the laboratory evaluation of ascites fluid, karyocyte count, Neu, Lym, ascitic fluid total protein (AFTP), ascitic fluid adenosine deaminase (AF ADA), glucose, chloride, and LDH were also reviewed. In addition, serum-ascites total protein gradient, ratio of chloride in serum and ascites, ratio of LDH in serum and ascites, platelet-to-lymphocyte ratio (PLR) in serum, and neutrophil-to-lymphocyte ratio (NLR) in both serum and ascites were evaluated.

### Diagnostic criteria

Three independent researchers with unknown etiology of ascites reviewed patient data to achieve a definitive diagnosis. If the diagnoses were inconsistent, the case was eliminated. Each case should have complete data and a clear diagnosis. The 317 patients were distributed under a well-established diagnosis basis and were divided into two groups: benign ascites group and malignant ascites group.

Patients in the malignant ascites group met at least one diagnosis criteria as follows: (1) the results of ascites fluid cytology were positive; (2) patients had a diagnostic peritoneal biopsy and obtained a positive result; (3) demonstration of a primary tumor or clinical evidence of tumor dissemination (e.g., lung metastases) and exclusion of other potential causes for the ascites.

The etiology of benign ascites included the following criteria: cirrhosis, nephrotic syndrome, spontaneous bacterial peritonitis, cardiac ascites, tuberculous peritonitis, pancreatic ascites; the well-established clinical criteria of the diagnosis of these diseases were described in previous studies 11-13. All the benign ascites were diagnosed by clinical and laboratory tests and without any tumor signs.

### Statistical analysis

All statistical analyses were performed using the SPSS software (version 21.0, IBM). All continuous variables were expressed as mean ± SD. Differences in continuous variables were assessed using the Student's t-test, while the chi-square test or Fisher's exact test was used for categorical data, as appropriate. To develop a diagnostic model, the 317 patients were randomly divided into two groups, a development dataset (60% of study subjects) or validation dataset (40% of study subjects). In the development group, all *P*-values less than 0.05 were considered for inclusion in univariate logistic regression analyses. Developing a more concise equation, receiver operating characteristic (ROC) analysis was performed (if the area under the ROC curve was ≥ 0.6) to determine which variables were used in further multivariable logistic regression analyses. The final diagnostic equation was then created by the β-coefficients of the multivariate logistic regression analysis, and the predicted performance was validated in the validation group. ROC curve analysis helped determine the optimal cut-off points for continuous variables based on their highest diagnostic accuracy. The sensitivity, specificity, positive predictive value (PPV), and negative predictive value (NPV) were calculated based on ROC analysis.

## Results

### Patient characteristics

In this study, clinical data from 384 patients with new-onset ascites were obtained for further study (Figure 1). The summary statistics of the etiology classification for the 317 included patients is shown in Table 1. They were first divided into two groups (benign and malignant) after stratifying according to disease type. A total of 94 patients were diagnosed with benign ascites. Liver causes accounted for 34.0% of cases, followed by peritoneal tuberculosis (13.9%) and congestive heart failure (12.8%). Among the 223 patients of the malignant ascites group, gastrointestinal tumor was the major cause of malignant ascites, which accounted for nearly 29.6% (66/223), followed by ovarian cancer, which accounted for 14.3% (32/223).

**Figure 2:**
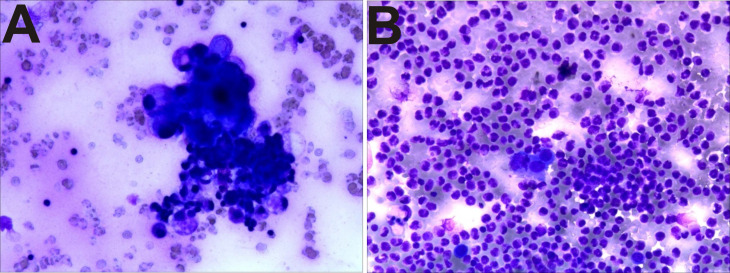
Pathological biopsy images showing (A) malignant ascites: adenocarcinoma cells, (B) benign ascites: neutrophils and mesothelial cells.

There were 102 cases of pathological cells positive in the 223 cases of malignant ascites (Figure 2A). The pathological cells of the benign group (94 cases) were all negative (Figure 2B), that is, the sensitivity of diagnosis of malignant ascites was 45.7%, specificity was 100%, and accuracy was 61.8%.

### Univariate analysis for discrimination between benign and malignant ascites

A total of 36 parameters were collected from the enrolled 317 patients, comprising two demographic features and 34 serum or AF laboratory parameters (Tables 2A, 2B). Comparisons between patients with benign and malignant ascites were made using the Student's t-test, and the results showed that 19 variables were significantly different. The malignant ascites and benign ascites (benign group: 47.5% female, 52.5% male vs. malignant group: 58.2% female, 41.8% male; *P >* 0.05) was not significantly different between sexes based on univariate analyses. In addition, the results demonstrated that patients with malignant ascites had a significantly older mean age (benign group 55.68 ± 17.22 vs. malignant group 62.57 ± 11.60, *P* < 0.05) than patients with benign ascites.

Among the serum laboratory parameters, WBC, TCh, hs-CRP, CA125, and CA19-9 were significantly higher than those assessed in the benign group. In contrast, the data analysis showed that the Cl levels in the benign group were significantly higher than those in the malignant group (Table 2A). In ascites, LDH levels were considerably higher in the malignant group. Moreover, Lym and ADA levels were also statistically different between the benign and malignant groups (Table 2B). For proportional indicators, serum-ascites total protein gradient was significantly higher in the benign group; in contrast, serum PLR was higher in the malignant group (Table 2B).

### Development of diagnostic models to discriminate malignant ascites from benign ascites

For analytic purposes, the enrolled patients were randomly divided into two groups: the development dataset (60% of study subjects) or validation dataset (40% of study subjects). The development dataset comprised 193 cases (59 benign ascites patients and 134 malignant ascites patients), and the validation dataset included 124 cases (35 patients with benign ascites and 89 with malignant ascites). No significant difference was found between mean age (60.50 ± 14.07 vs. 59.45 ± 15.13 years; *P* = 0.856) and distribution of males and females (Males: Females, 87:106 vs. 52:72; *P* = 0.846) between the development and validation groups. Then, the development dataset was used to create a diagnostic model. The list of screened variables (*P* < 0.05) was selected as a candidate marker to differentiate malignant ascites from benign ascites using univariate logistic regression analysis (Table 3). Further, parameters with higher diagnostic values (AUC ≥ 0.6) such as age, WBC, TCh, hs-CRP, AF ADA, and AF LDH were selected as diagnostic scoring model markers in the development set. After the multivariable binary logistic regression analysis was applied, the WBC count was eliminated. Thus, age, TCh, hs-CRP, AF ADA, and AF LDH remained for the first model in the development set. Furthermore, two vital markers (CA125 (AUC = 0.597) and CA199 (AUC = 0.594)) were also incorporated into the predictive model (the second model) because of their significance in clinical practice (Table 4).

The following mathematic models were used to estimate the probability of developing malignant ascites (a higher score implies a higher likelihood of malignant ascites). The predictive performance of the model was evaluated by AUC.

5-markers risk score = 1/[1 + e^-(-4.909 + 0.037 × age + 0.638 × TCh + 0.011 × hs-CRP - 0.088 × ADA + 0.006 × LDH)^]

7-markers risk score = 1/[1 + e^-(-4.985 + 0.036 × age + 0.515 × TCh + 0.001 × hs-CRP - 0.083 × ADA + 0.005 × LDH + 0.001 × CA125 + 0.002 × CA19-9)^]

• Code used for the equations:

1. P, predictive value;

2. e, natural logarithm.

**Figure 3:**
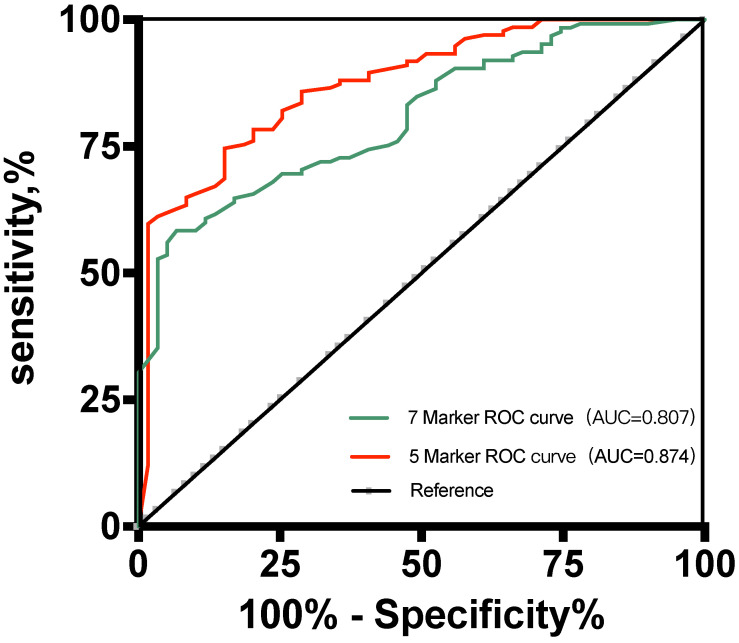
Receiver operating characteristic curves for the two predictive models for the development set.

The results showed that the 5-marker predictive model reached an AUC of 0.874 (95% confidence interval [CI], 0.822-0.926) (Figure 3). Under the predicted threshold of 0.83, the sensitivity, specificity, PPV, NPV, PLR, NLR, and accuracy were 84.7%, 88.8%, 92.9%, 76.9%, 7.56%, 0.17%, and 87.6%, respectively, in the 5-marker scoring system. Moreover, we analyzed the performance of single parameters and 7-marker model in discriminating these two conditions. As expected, the AUC of the single variable and 7-marker model (AUC = 0.807) was lower than the AUC of the 5-marker model (Figure 3).

### Validation of the diagnostic model

**Figure 4:**
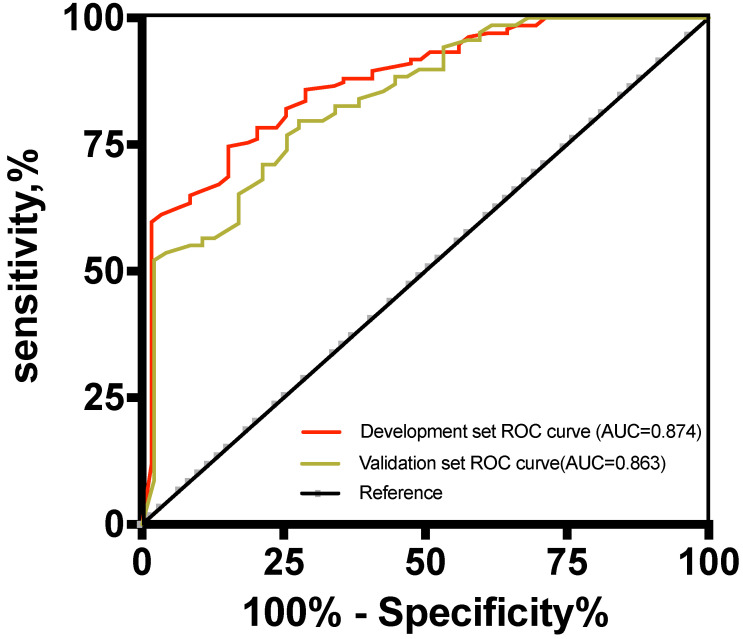
Receiver operating characteristic curves for the development and validation datasets.

**Figure 5:**
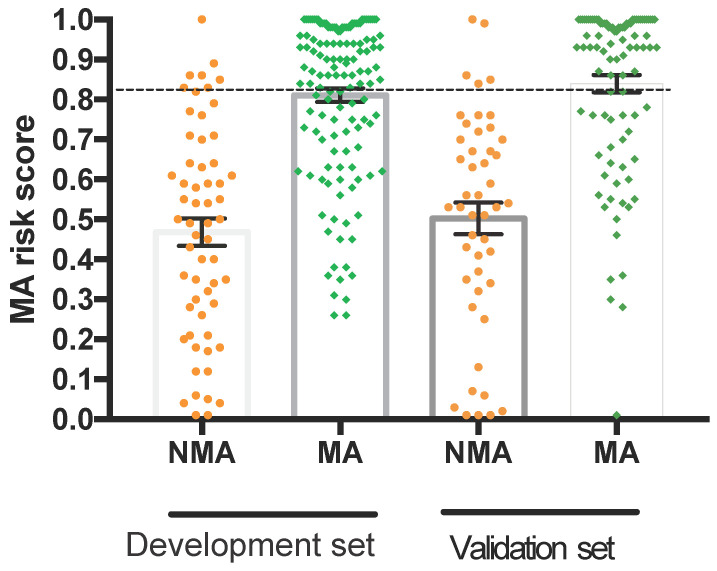
Dot plots of the 5-marker malignant ascites risk score in differentiating benign and malignant ascites by datasets. Error bars indicate the mean and standard error of the mean. The dotted line represents the cut-off value for predicting malignant ascites at 0.83. NMA: non-malignant ascites; MA: malignant ascites.

The risk score of the 5-marker predictive model was validated using the validation dataset. ROC analysis showed that the AUC of the predictive model was 0.863 (95% CI, 0.771-0.887) (Figure 4), which was not significantly different between the two datasets. In the validation set, the performance parameters were as follows: sensitivity, 80.9%; specificity, 82.6%; PPV, 92.3%; NPV, 80.5%; PLR, 4.65; NLR, 0.23; the ability to correctly classify the two conditions was 81.5% with a cut-off point of 0.83. All validity indexes listed in the results of the training set and validation set for the 5-marker predictive model were similar, and the predictive model showed good diagnostic efficiency in both datasets. As shown in Figure 4, when the cut-off point was 0.83, the proportions of false-negative malignant ascitic patients and false-positive benign ascitic patients were 15/134 and 9/59 in the development set and 17/89 and 6/35 in the validation set, respectively (Figure 5).

## Discussion

Malignant ascites is a common complication associated with a wide variety of neoplasms, including liver, pancreatic, gastric, colorectal, and ovarian cancers. Stukan et al. 14 reported that malignant ascites is a sign of advanced cancer and poor prognosis, requiring timely, appropriate treatment. Therefore, the first and foremost goal of this study was to facilitate the early diagnosis of ascites to improve the short mean survival duration of patients with malignant ascites 15. Traditionally, differential diagnosis can be made based on certain specific findings such as AF cytology and diagnostic peritoneal biopsy. Unfortunately, these methods have limitations such as invasiveness and limited diagnostic efficacy 16. In this study, the sensitivity of cytology was 45.7%, and this result was also unsatisfactory.

To date, several serum or AF markers have been studied, including vascular endothelial growth factor 17, endostatin 18, and several cytokines 19. Despite the higher diagnostic value, these new markers were inaccessible in public clinical settings. Moreover, the value of using a single parameter to distinguish these two conditions is absolutely limited in clinical practice because of low sensitivity or specificity, while the combination of several markers to construct a mathematical model will significantly improve the diagnostic accuracy. Therefore, 36 parameters of 317 enrolled patients were collected with nearly all forms of ascites. A 5-marker predictive model was then established using multivariate regression analysis in the development set, while the diagnostic performance was evaluated in the validation set. To our knowledge, this is the first study to develop a simple, user-friendly predictive model based on the most ordinary clinical parameters to identify malignant ascites.

All easily available clinical and laboratory features were analyzed in this study. The serological examination is the basic examination that all patients will undergo when admitted to the hospital. On univariate analysis of the serum laboratory features in our study, TCh and hs-CRP were included as features favoring malignant ascites. In recent years, the relationship between inflammation and tumors has gradually become a research focus 20. CRP is an acute-phase reactant that increases during acute or chronic inflammation and infections 21, 22. hs-CRP is more sensitive than CRP as an inflammatory marker and is known to be elevated in chronic liver diseases and spontaneous bacterial peritonitis. Abdel-Razik et al. 1 studied 398 consecutive patients with ascites and found that CRP was a useful marker for discriminating between malignancy-related and benign ascites. Wiese et al. 23 showed that proinflammatory (hs-CRP) markers can predict the severity of liver disease, cardiac and hemodynamic changes, and long-term survival outcomes. However, little research has been conducted on the role of hs-CRP in distinguishing between benign and malignant ascites. The results of our present study suggest that the diagnostic significance of hs-CRP for identifying malignant ascites was superior to that of CRP. NLR and PLR also reflect the level of systemic inflammation in the body, which is significantly related to cell destruction caused by tumors 24. Previous studies have confirmed that NLR and PLR help predict the prognosis of tumors 25-27, but few studies have explored the function of NLR and PLR in diagnosing malignant ascites. We first found significant differences in PLR between benign and malignant ascites, although this was not included in this predictive model.

Abnormal lipid metabolism has been reported in various studies to be closely related to the development of tumors 28 and will increase the risk of cancers 29, 30. In addition, Banerjee et al. 31 showed that cholesterol in AF, as well as in serum, can be used as diagnostic markers to evaluate the nature of ascites. A study conducted by Farwell et al. 32 also suggested that increased levels of serum cholesterol indicate a higher risk for total and high-grade prostate cancer. The cholesterol in serum is a valuable parameter for patients diagnosed in our study, similar to previous findings 19, 33. The underlying causes of cholesterol elevation in serum were unidentified, which merit further investigation, but this should not confine its scientific value.

Abdominal paracentesis is likely the fastest and most cost-effective way to diagnose the cause of ascites. The clinical diagnostic efficiency of AF ADA and LDH in the etiological diagnosis of unknown origin ascites is reportedly satisfactory 34-36. There is a large body of work on the function of tumor markers, which showed that it helped identify malignant ascites 6, 27, 37. However, AFP and CEA levels were not significant (*P* > 0.05) in our study, and they were not included in the predictive model. The difference between our study and previous studies may be attributed to the different causes of benign ascites in the previous study and our study. The results showed that the percentage of liver cirrhosis and miscellaneous lesions (34.04%, 39.36%, respectively) in our study was higher than that in previous studies (16.12%, 9.68%) 38. Liver cirrhosis also leads to elevated AFP levels. Previous studies also demonstrated that the CEA of AF does not seem specific enough to diagnose malignancy-related ascites 39, 40. Similar to our results, the sensitivity and specificity of CEA were 97% and 50%, respectively. The results of our study also showed that the 7-marker model has a lower diagnostic ability than the 5-marker model, which may be related to the reason mentioned above. Although a significant increase in CA125 and CA19-9 is considered a valuable indicator for cancer diagnosis 41, 42, with continuous science development, many scholars have found that CA125 and CA199 has a varying degree of increase and significance in the diagnosis of non-malignant diseases, such as heart failure 43, active tuberculosis 44, inflammatory bowel disease 45, liver cirrhosis 46, and other diseases. In addition, previous studies reported no significant differences in laboratory values, such as WBCs, platelets, and some other variables between malignant and benign ascites, which were similar to those of our results 47.

Numerous studies have attempted to identify a more effective method for the early diagnosis of malignant ascites. However, to date, no sensitive and convenient method for differentiating ascites has been determined. Thus, a combination of the selected valuable parameters to establish a mathematical model may help solve this problem. In 2000, Alexandrakis et al. 8 reported a scoring model including TP, LDH, TNF-α, C4, and haptoglobin, with an accuracy of 89% in the overall date and 70% in the cross-validation date. Tian et al. 48 also calculated scores for diagnosing malignant ascites, including innovative features-effusion CEA, effusion tumor cells (ETC), and ETC cluster count, with an AUC of the training dataset and validation dataset of 0.939 and 0.948, respectively. We also developed a predictive score model for the same purpose. The indicators included in our study are particularly common variables and highly clinically useful. In contrast, with similar diagnostic power, previous models may include disadvantages of high cost, the need for additional examinations, and trained personnel. Moreover, results using this predictive model for a patient with unknown etiology of ascites can be determined easily by entering each value into the risk score formula by calculation.

Our study has several limitations. First, some potentially important markers for malignant diagnoses, such as erythrocyte sedimentation rate (ESR) and AF CEA, were not evaluated in our study, as only some included patients underwent that test. Nevertheless, previous studies have shown that ESR showed no significant difference between benign and malignant ascites 49. Second, this is a single-center, retrospective study with a limited number of patients. The prevalence of ascites in this study may not reflect the prevalence of ascites in the Chinese population. Therefore, further studies with a larger sample size from multiple centers are needed to validate this predictive model. Thus, it is necessary to reassess the predictive models if they are used in a different population.

In summary, we have established a user-friendly and reliable 5-marker predictive model for classifying ascites into subtypes (malignant or benign). We believe that this simple mathematical model could be a useful diagnostic aid to distinguish malignant from benign ascites in clinical practice.

## Figures and Tables

**Table 1:** The aetiology of ascites in the patients enrolled in our study

Aetiology	Benign group (n=94)	Malignant group (n=223)	Total (n=317)
Cirrhosis	32	0	32
Cardiac ascites	12	0	12
Tuberculous peritonitis	13	0	13
Miscellaneous	37	0	37
Liver Cancer	0	31	31
Pancreatic Cancer	0	22	22
Gastric Cancer	0	44	44
Colorectal Cancer	0	24	24
Ovarian Cancer	0	32	32
Metastatic peritoneal carcinoma	0	16	16
Other Cancers	0	54	54

**Table 2A:** Demographic and serum laboratory features of patients with benign and malignant ascites

Demographic and laboratory features of patients	Benign group (n=94)	Malignant group (n=223)	*P* value
Age, y	54.48±18.32	62.07±12.63	0.006
Sex, male: female	45:49	111:112	0.168
WBCs (×10^9^/L)	6.20±3.38	7.02±2.84	0.006
Neu (×10^9^/L)	4.55±2.91	8.103±27.63	0.228
Lym (×10^9^/L)	1.02±0.71	2.04±8.97	0.294
RBC (×10^12^/L)	3.71±1.01	3.83±0.73	0.08
Hb (g/L)	108.63±27.53	113.11±19.93	0.043
RDW-SD	50.36±9.93	48.65±9.92	0.105
PLT (×10^6^/L)	199.48±117.82	283.65±146.40	P<0.001
PDW	12.71±2.85	12.13±2.43	0.070
ALP (U/L)	151.17±217.38	131.47±150.71	0.443
TP (g/L)	63.26±7.1	63.47±7.52	0.991
ALB (g/L)	33.91±5.38	35.19±4.87	0.106
Cl (mmol/L)	103.54±5.94	102.71±4.98	0.032
TCh (mmol/L)	3.42±1.00	4.31±1.50	P<0.001
hs-CRP (mg/L)	39.41±42.76	53.70±51.52	P<0.001
CRP (mg/L)	34.46±36.48	47.38±47.10	0.043
LDH (U/L)	272.30±153.91	330.58±244.66	0.022
AFP (ng/mL)	2661.91±13586.12	1535.51±9741.86	0.094
CA125 (U/L)	513.91±546.45	1159.46±2112.09	P<0.001
CA19-9 (U/L)	35.11±143.57	1242.98±5775.03	0.028
CEA (ng/mL)	34.03±145.00	102.96±661.30	0.581

CRP, hypersensitivity C-reactive protein; CRP, C-reactive protein; LDH, lactate dehydrogenase; AFP, alpha-fetoprotein assay; CA125, cancer antigen 125; CA19-9, cancer antigen 19-9; CEA, carcinoembryonic antigen. *Data are presented as mean (±) standard deviation.

**Table 2B:** Ascitic fluid laboratory features and other parameter of patients with benign and malignant ascites

Demographic and laboratory features of patients	Benign group (n=94)	Malignant group (n=223)	*P* value
Karyocyte count	0.94±1.71	1.00±1.48	0.642
Neu%	15.46±19.96	20.19±17.64	0.04
Lym%	77.42±23.00	66.58±19.79	P<0.001
AFTP (g/L)	30.00±1703	36.74±14.36	0.004
ADA (U/L)	16.72±23.10	8.98±10.12	0.02
Glu (mmol/L)	8.22±11.08	6.37±2.31	0.112
Cl (mmol/L)	126.51±168.00	107.32±5.55	0.36
LDH (U/L)	186.73±189.43	424.44±507.28	*P*<0.001
Serum-ascites total protein gradient	32.44±23.08	26.74±14.74	0.009
Serum-to-ascites LDH ration	2.54±6.65	1.04±0.87	0.583
Serum-to-ascites Cl ration	0.90±0.25	4.61±45.74	0.688
NLR of serum	5.37±3.76	6.52±2.34	0.028
NLR of ascites	7.07±6.84	8.01±3.32	0.600
PLR of serum	302.83±234.91	348.34±223.54	P<0.01

Neu, neutrophil; Lym, lymphocyte; AFTP, ascitic fluid total protein (AFTP); ADA, adenosine deaminase (ADA); Glu, glucose; CL, chloride; LDH, lactate dehydrogenase; NLR, neutrophils-to-lymphocyte ratio; PLR, platelet-lymphocyte ratio. *Data are presented as mean (±) standard deviation.

**Table 3:** Univariate logistic regression analysis of significant variables in the development set

Variable	B	S. E	Wald	*P* value	OR (95%CI)
Age	0.036	0.012	9.422	0.002	1.036 (1.013-1.060)
WBCs	0.169	0.063	7.128	0.008	1.184 (1.046-1.341)
Hb	0.015	0.007	4.023	0.325	1.015 (1.000-1.030)
PLT	0.005	0.001	11.676	0.383	1.005 (1.002-1.008)
Cl	-0.073	0.034	4.447	0.020	0.930 (0.869-0.995)
TC	0.688	0.189	13.232	<0.001	1.990 (1.373-2.883)
hs-CRP	0.012	0.004	8.941	0.003	1.012 (1.004-1.020)
CRP	0.009	0.005	4.262	0.454	1.009 (1.000-1.018)
LDH	0.002	0.001	3.538	0.414	1.002 (1.000-1.004)
CA125	0.001	0.001	5.939	0.011	1.001 (1.000-1.001)
CA199	0.003	0.001	5.432	0.003	1.674 (1.000-1.005)
AF Neu%	0.021	0.010	4.230	0.060	1.021 (1.001-1.042)
AF lym%	-0.034	0.010	12.902	<0.001	0.966 (0.949-0.985)
AF TP (g/L)	0.033	0.011	10.250	0.441	1.035 (1.013-1.056)
AF ADA (U/L)	-0.027	0.010	6.607	<0.001	0.974 (0.954-0.994)
AF LDH (U/L)	0.003	0.001	13.776	<0.001	1.003 (1.001-1.005)
Serum-ascites total protein gradient	-0.032	0.010	9.823	<0.001	0.969 (0.950-0.988)
Serum NLR	0.064	0.037	3.023	0.082	1.066 (0.992-1.146)
Serum PLR	0.002	0.001	4.869	0.027	1.002 (1.000-1.003)

AF: ascitic fluid.

**Table 4:** Diagnostic values of the variables used to differentiate benign and malignant ascites in the development set

Variable	AUC (95%CI)	*P* value (logistic regression)
Age	0.614 (0.520-0.705)	0.036
WBC	0.643 (0.552-0.733)	0.577
Cl	0.406 (0.314-0.497)	0.966
TC	0.689 (0.605-0.773)	0.023
hs-CRP	0.616 (0.535-0.698)	0.016
CA125	0.597 (0.535-0.695)	0.126
CA199	0.594 (0.512-0.676)	0.178
lym% (ascites)	0.287 (0.201-0.373)	0.633
ADA (U/L) (ascites)	0.624 (0.520-0.728)	<0.001
LDH (U/L) (ascites)	0.712 (0.634-0.789)	0.005
Serum-ascites total protein gradient	0.401 (0.305-0.496)	0.05
PLR	0.595 (0.476-0.697)	0.640

AUC: Area under ROC curve; Bold values signify seven diagnostic values among area under the ROC ≥0.6.

## Abbreviations

AF: ascitic fluid
ROC: receiver operating characteristic
hs-CRP: hypersensitivity C-reactive protein
WBCs: white blood cells
Neu: neutrophil
Lym: lymphocyte
RBCs: red blood cells
PDW-SD: red blood cell distribution width-standard deviation
PDW: platelet distribution width
ALP: alkaline phosphatase
TP: total protein
Tch: total cholesterol
LDH: lactate dehydrogenase
AFP: alpha-fetoprotein assay
CA125: cancer antigen 125
CA19-9: cancer antigen 19-9
CEA: carcinoembryonic antigen
AFTP: ascitic fluid total protein
ADA: adenosine deaminase
PLR: platelet-to-lymphocyte ratio
NLR: neutrophil-to-lymphocyte ratio
VEGF: vascular endothelial growth factor
NMA: non-malignant ascites
MA: malignant ascites
